# Assessment of Herpes Zoster Risk Among Recipients of COVID-19 Vaccine

**DOI:** 10.1001/jamanetworkopen.2022.42240

**Published:** 2022-11-16

**Authors:** Idara Akpandak, D. Claire Miller, Yuwei Sun, Benjamin F. Arnold, J. Daniel Kelly, Nisha R. Acharya

**Affiliations:** 1 F.I. Proctor Foundation, University of California, San Francisco, San Francisco; 2 Department of Ophthalmology, University of California, San Francisco, San Francisco; 3 Department of Epidemiology and Biostatistics, University of California, San Francisco, San Francisco; 4 Institute for Global Health Sciences, University of California, San Francisco, San Francisco

## Abstract

**Importance:**

Herpes zoster infection after COVID-19 vaccination has been reported in numerous case studies. It is not known whether these cases represent increased reporting or a true increase in risk.

**Objective:**

To assess whether COVID-19 vaccination is associated with an increased risk of herpes zoster infection.

**Design, Setting, and Participants:**

This cohort study used a self-controlled risk interval (SCRI) design to compare the risk of herpes zoster in a risk interval of 30 days after COVID-19 vaccination or up to the date of the second vaccine dose with a control interval remote from COVID-19 vaccination (defined as 60-90 days after the last recorded vaccination date for each individual, allowing for a 30-day washout period between control and risk intervals). A supplemental cohort analysis was used to compare the risk of herpes zoster after COVID-19 vaccination with the risk of herpes zoster after influenza vaccination among 2 historical cohorts who received an influenza vaccine in the prepandemic period (January 1, 2018, to December 31, 2019) or the early pandemic period (March 1, 2020, to November 30, 2020). Data were obtained from Optum Labs Data Warehouse, a US national deidentified claims-based database. A total of 2 039 854 individuals who received any dose of a COVID-19 vaccine with emergency use authorization (BNT162b2 [Pfizer-BioNTech], mRNA-1273 [Moderna], or Ad26.COV2.S [Johnson & Johnson]) from December 11, 2020, through June 30, 2021, were eligible for inclusion. Individuals included in the SCRI analysis were a subset of the COVID-19–vaccinated cohort who had herpes zoster during either a risk or control interval.

**Exposures:**

Any dose of a COVID-19 vaccine.

**Main Outcomes and Measures:**

Incident herpes zoster, defined by *International Statistical Classification of Diseases and Related Health Problems, Tenth Revision *codes and a prescription of a new antiviral medication or a dose increase in antiviral medication within 5 days of diagnosis.

**Results:**

Among 2 039 854 individuals who received any dose of a COVID-19 vaccine during the study period, the mean (SD) age was 43.2 (16.3) years; 1 031 149 individuals (50.6%) were female, and 1 344 318 (65.9%) were White. Of those, 1451 patients (mean [SD] age, 51.6 [12.6] years; 845 [58.2%] female) with a herpes zoster diagnosis were included in the primary SCRI analysis. In the SCRI analysis, COVID-19 vaccination was not associated with an increased risk of herpes zoster after adjustment (incidence rate ratio, 0.91; 95% CI, 0.82-1.01; *P* = .08). In the supplementary cohort analysis, COVID-19 vaccination was not associated with a higher risk of herpes zoster compared with influenza vaccination in the prepandemic period (first dose of COVID-19 vaccine: hazard ratio [HR], 0.78 [95% CI, 0.70-0.86; *P* < .001]; second dose of COVID-19 vaccine: HR, 0.79 [95% CI, 0.71-0.88; *P* < .001]) or the early pandemic period (first dose of COVID-19 vaccine: HR, 0.89 [95% CI, 0.80-1.00; *P* = .05]; second dose: HR, 0.91 [95% CI, 0.81-1.02; *P* = .09]).

**Conclusions and Relevance:**

In this study, there was no association found between COVID-19 vaccination and an increased risk of herpes zoster infection, which may help to address concerns about the safety profile of the COVID-19 vaccines among patients and clinicians.

## Key Points

**Question:**

Is there an increased risk of herpes zoster infection after COVID-19 vaccination?

**Findings:**

In this cohort study of 2 039 854 individuals who received a COVID-19 vaccine included in a US health care claims database, a self-controlled risk interval analysis revealed that the incidence rate ratio of herpes zoster after COVID-19 vaccination was 0.91. A supplementary cohort analysis found no increased risk of herpes zoster after COVID-19 vaccination compared with influenza vaccination in the prepandemic and early pandemic period.

**Meaning:**

These findings suggest COVID-19 vaccination is not associated with an increased risk of herpes zoster, which may help to address concerns about the safety profile of COVID-19 vaccines.

## Teaser

This cohort study uses data from a US national claims database to assess whether COVID-19 vaccination is associated with herpes zoster infection.

## Introduction

The rapid production and distribution of COVID-19 vaccines have been key measures in reducing the burden of disease associated with SARS-CoV-2 infection. As millions of US residents complete their COVID-19 primary immunization series and receive additional doses or booster injections to maintain adequate immunity, there remains a need for vaccine safety monitoring. Surveillance of vaccine safety in clinical practice is important after licensure when a vaccine is administered to large numbers of the general population, including subgroups such as immunocompromised individuals who were not included in initial clinical trials.^1,2^

Due to the world-altering nature of COVID-19, postvaccination adverse events have been a focus of both scientific and public attention.^3,4,5^ Multiple centers in the US and abroad have published case reports of herpes zoster after the first and second doses of a COVID-19 vaccine.^6,7,8,9^ Herpes zoster, also known as shingles, is caused by a reactivation of latent varicella zoster virus and manifests as a blistering dermatomal rash that can lead to long-term pain and reduced quality of life.^10,11^ It is unknown whether these case reports represent increased reporting of randomly occurring herpes zoster cases given the increased attention on the COVID-19 vaccine or a true increase in risk. Although these case reports could not definitively establish an association between COVID-19 vaccination and herpes zoster, many of the reports postulated that an association may exist. This ambiguity has generated uncertainty among physicians and concern among patients, contributing to vaccine hesitancy.^12^ Characterization of this risk is important to vaccine safety monitoring and to inform ongoing vaccination efforts. Therefore, we performed this claims-based study to assess whether there was an increased risk of herpes zoster after COVID-19 vaccination.

## Methods

This study was approved by the institutional review board of the University of California, San Francisco, with a waiver of consent due to having no more than minimal risk to participants and using deidentified claims data with no contact information on participants available. The study was conducted in adherence to the tenets of the Declaration of Helsinki.^13^ This study followed the Strengthening the Reporting of Observational Studies in Epidemiology (STROBE) reporting guideline for cohort studies.

### Data Source

This retrospective cohort study used deidentified health care claims data from Optum Labs Data Warehouse (OLDW; Optum Labs).^14^ The OLDW contains administrative claims and electronic health record information for commercial and Medicare Advantage enrollees, including medical and pharmacy claims, laboratory results, and enrollment information. Medical claims include diagnosis codes (ie, *International Statistical Classification of Diseases and Related Health Problems, Tenth Revision* [*ICD-10*] codes, *Current Procedural Terminology* [*CPT*] codes, and Healthcare Common Procedure Coding System [HCPCS] codes), dates of service, and specialty codes of health care professionals. Pharmacy claims data include National Drug Code (NDC), brand name, generic name, quantity, days’ supply, drug strength, drug administration route, and the date the prescription was filled. The database includes a diverse sample of individuals by age, race and ethnicity, and geography throughout the US. Geographically, the OLDW has a higher proportion of enrollees in the southern and central regions of the US. In December 2020, the month COVID-19 vaccines were first administered in the US, there were approximately 14 million individuals enrolled with both medical and pharmacy coverage in the OLDW.

### Study Population

Individuals who received any dose of a COVID-19 vaccine with emergency use authorization from the US Food and Drug Administration (BNT162b2 [Pfizer-BioNTech], mRNA-1273 [Moderna], or Ad26.COV2.S [Johnson & Johnson]) from December 11, 2020, through June 30, 2021, were eligible to be included in the study. Eligible participants were further required to be continuously enrolled in both medical and pharmacy coverage from 270 days before the date of first recorded COVID-19 vaccine dose (index date) through July 31, 2021, to allow for determination of baseline characteristics and herpes zoster cases after vaccination. Individuals with vaccination records inconsistent with guidance from the Centers for Disease Control and Prevention (CDC) at the time of the study (eg, excess doses, receipt of a vaccine before the vaccine’s date of emergency use authorization, or receipt of a vaccine before age eligibility) were excluded. COVID-19 vaccines were identified by the presence of a CPT or HCPCS code in the medical claims database or by an 11-digit NDC or drug name text search in the pharmacy claims database (eTable 1 in the Supplement). Individuals with a previous diagnosis of herpes zoster (identified by *ICD-10* code B02.xx) in the 270 days before the index date were excluded.

### Outcome Identification

Individuals who received a COVID-19 vaccine were evaluated for herpes zoster infection in the 30 days after receiving a first dose (for BNT162b2, mRNA-1273, and Ad26.COV2.S) or second dose (for vaccines with 2-dose regimens [BNT162b2 and mRNA-1273] only) of the vaccine or, in the case that a second dose was administered before 30 days had elapsed, up to the date of the second dose. Herpes zoster diagnoses were identified during these periods using *ICD-10* code B02.xx in the primary or secondary position in a medical claim. Both a herpes zoster diagnosis and a prescription for a systemic antiviral medication (acyclovir, valacyclovir, or famciclovir) were required to qualify as a herpes zoster outcome event. For patients who were not receiving antiviral medications at the time of diagnosis, we required a new antiviral prescription within 5 days after the first herpes zoster diagnosis. For patients who were receiving antiviral medications at the time of diagnosis, we required either a dose increase in the medication or the addition of an oral or ophthalmic steroid medication within 5 days after the first herpes zoster diagnosis (eTable 2 in the Supplement). Antiviral dose increases were identified using the methods described in the eAppendix and eTable 3 in the Supplement.

### Self-controlled Risk Interval Analysis

**Figure 1.**
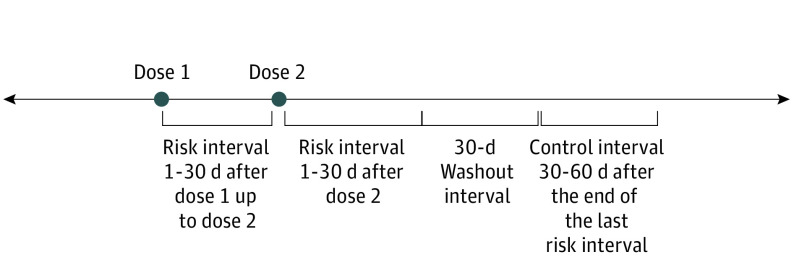
Illustration of Self-controlled Risk Interval Design Dose 1 and dose 2 represent the first and second doses of a COVID-19 vaccine (the BNT162b2 and mRNA-1273 vaccines have a 2-dose regimen, and the Ad26.COV2.S vaccine has a 1-dose regimen). The second dose (of the BNT162b2 and mRNA-1273 vaccines) was typically administered 21 to 28 days after the first dose. Individuals were observed for herpes zoster infection in the risk intervals (after dose 1 for all 3 vaccines and after dose 2 for the BNT162b2 and mRNA-1273 vaccines) and in the control interval (30-60 days after the final risk interval ended), leaving 30 days for the washout interval. In this design, the risk of herpes zoster in the risk interval was compared with the risk of herpes zoster in the control interval within each individual. Risk intervals after each dose were modeled together and separately.

Due to incomplete capture of COVID-19 vaccination records in US claims-based databases as well as potentially large differences in the behaviors and health status of individuals who chose to remain unvaccinated vs receive a vaccine,^15^ a self-controlled risk interval (SCRI) design was used as the primary study design. This design involved comparing the incidence of herpes zoster in an exposed risk interval immediately after COVID-19 vaccination with a predefined unexposed control interval remote from vaccination within the same person. The entire eligible COVID-19–vaccinated population was evaluated for the herpes zoster outcome, but only patients who had an incident herpes zoster event in the risk or control interval were included in the risk estimation.^16^ This design inherently controlled for time-invariant factors, such as age, demographic characteristics, chronic diseases, and long-term medication use, and has been used extensively to study vaccine safety.^17,18^ We defined the control interval as 60 to 90 days after the last recorded vaccination date for each patient, allowing for a 30-day washout period between the control and risk intervals.^16^ A schematic of the SCRI timeline within a patient is shown in Figure 1. Herpes zoster outcomes were identified during the control interval using the same criteria described in the previous section.

Subgroup analyses of the population included in the SCRI analysis were also conducted by age (<50 years or ≥50 years), immunocompromised status at baseline (determined based on history of HIV or AIDS, cancer, solid organ transplant, systemic corticosteroid use, or immunosuppressive medication use [eTable 5 and eTable 7 in the Supplement]), and COVID-19 vaccine type (BNT162b2, mRNA-1273, or Ad26.COV2.S).

### Supplemental Cohort Analysis

A cohort study design was used to conduct a supplemental analysis. The COVID-19–vaccinated cohort included all eligible individuals, regardless of whether they experienced the herpes zoster outcome. The COVID-19–vaccinated cohort was compared with 2 historical cohorts comprising individuals who received an influenza vaccine from January 1, 2018, to December 31, 2019 (prepandemic group), or from March 1, 2020, to November 30, 2020 (early pandemic group), to understand whether COVID-19 vaccination was associated with a higher than expected risk of herpes zoster. Two cohorts who received influenza vaccines were used for comparison due to concerns that health care use may have differed between pre–COVID-19 and COVID-19 periods.^19^ Details on the methods used for this design are available in the eAppendix and eTables 4 to 9 in the Supplement.

### Statistical Analysis

For the SCRI analysis, conditional Poisson regression models were used to estimate incidence rate ratios (IRRs) and corresponding 95% CIs comparing the risk of herpes zoster in the risk interval with the risk of herpes zoster in the control interval. An offset of the natural log of the entire length of the observed interval was used to account for unequal control and risk interval lengths. Risk intervals after first and second doses were modeled together and separately.

Statistical analyses were performed using R software, version 4.1.0 (R Foundation for Statistical Computing). Two-sided hypothesis tests were used, and *P* < .05 was considered statistically significant.

## Results

Among 2 039 854 individuals who received any dose of a COVID-19 vaccine during the study period, the mean (SD) age was 43.2 (16.3) years; 1 031 149 individuals (50.6%) were female, 1 008 543 (49.4%) were male, and 162 (<0.1%) were of unknown sex; 139 826 (6.9%) were Asian, 141 582 (6.9%) were Black, 205 463 (10.1%) were Hispanic, 1 344 318 (65.9%) were White, and 208 665 (10.2%) were of unknown race and/or ethnicity. Of those who received any dose of a COVID-19 vaccine during the study period, 1451 individuals had herpes zoster infection in either a risk or control interval and were included in the SCRI analysis. Of those, 845 patients (58.2%) were female, 606 (41.8%) were male, 99 (6.8%) were Asian, 80 (5.5%) were Black, 131 (9.0%) were Hispanic, 1026 (70.7%) were White, and 115 (7.9%) were of unknown race and/or ethnicity; the mean (SD) age was 51.6 (12.6) years.

The baseline demographic characteristics, comorbidities, and health care use of eligible individuals who received any dose of a COVID-19 vaccine and patients included in the primary SCRI analysis are shown in Table 1. The most prevalent comorbidities among individuals included in the SCRI analysis were diabetes (157 patients [10.8%]), autoimmune disease (144 patients [9.9%]), and cardiovascular disease (92 patients [6.3%]). At the time of the first recorded vaccine dose, 95 patients (6.5%) were receiving systemic corticosteroids or other immunosuppressant medications. A total of 2575 COVID-19 vaccine doses (1396 first doses [54.2%] and 1179 second doses [45.8%]) were administered within this population, with the most administered vaccine type being BNT162b2 (1437 doses [55.8%]) followed by mRNA-1273 (1051 doses [40.8%]) and Ad26.COV2S (87 doses [3.4%]). The mean (SD) observation time after receipt of a dose was 27.8 (3.5) days. Additional characteristics of the COVID-19 vaccine doses are shown in Table 2.

**Table 1.** Characteristics of the Eligible Study Population and Those Included in the Self-controlled Risk Interval Analysis

Characteristic	Patients, No. (%)	
	Eligible COVID-19–vaccinated population (n = 2 039 854)	SCRI population (n = 1451)^a^
Age, y		
Mean (SD)	43.2 (16.3)	51.6 (12.6)
Median (IQR)	44.0 (31.0-56.0)	52.0 (42.0-60.0)
Sex		
Female	1 031 149 (50.6)	845 (58.2)
Male	1 008 543 (49.4)	606 (41.8)
Unknown	162 (<0.1)	0
Race and ethnicity^b^		
Asian	139 826 (6.9)	99 (6.8)
Black	141 582 (6.9)	80 (5.5)
Hispanic	205 463 (10.1)	131 (9.0)
White	1 344 318 (65.9)	1026 (70.7)
Unknown	208 665 (10.2)	115 (7.9)
Health care use		
Ambulatory visit count		
Mean (SD)	8.4 (11.5)	11.7 (14.1)
Median (IQR)	5.0 (2.0-10.0)	7.0 (3.0-15.0)
Inpatient visit ever	227 146 (11.1)	224 (15.4)
Medical history		
Asthma	85 468 (4.2)	74 (5.1)
Autoimmune disease	108 250 (5.3)	144 (9.9)
Cancer	52 850 (2.6)	74 (5.1)
Cardiovascular disease	65 735 (3.2)	92 (6.3)
Chronic kidney disease	40 655 (2.0)	66 (4.5)
Chronic lung disease	34 663 (1.7)	48 (3.3)
Diabetes (any type)	154 694 (7.6)	157 (10.8)
HIV or AIDS	5130 (0.3)	<11 (<0.8)
Solid organ transplant	3929 (0.2)	<11 (<0.8)
Recent COVID-19 infection^c^^,^^d^	20 482 (1.0)	18 (1.2)
Received herpes zoster vaccine	160 380 (7.9)	65 (4.5)
Medication use^d^		
Systemic corticosteroids	10 788 (0.5)	25 (1.7)
Other immunosuppressants	36 998 (1.8)	70 (4.8)
Antivirals	14 300 (0.7)	<11 (<0.8)

Abbreviation: SCRI, self-controlled risk interval.

^a^ Patients included in the SCRI analysis had herpes zoster infection in either a risk or control interval.

^b^ Due to rounding, the percentages for the SCRI population sum to 99.9%.

^c^ Recent COVID-19 infection was defined as infection within 30 days before the start of the risk interval up to the event or censor date.

^d^ For patients who received a COVID-19 vaccine, recent COVID-19 infection and medication use were measured at the dose level; status at the first recorded dose was used to report the frequencies and percentages in this table.

**Table 2.** Characteristics of Eligible COVID-19 Vaccine Doses and Doses Included in the Self-controlled Risk Interval Analysis

Characteristic	COVID-19 vaccine doses, No. (%)	
	Eligible (n = 3 551 451)	SCRI analysis (n = 2575)^a^
Type of vaccine		
BNT162b2	2 157 623 (60.8)	1437 (55.8)
mRNA-1273	1 244 403 (35.0)	1051 (40.8)
Ad26.COV2.S	149 425 (4.2)	87 (3.4)
Dose administered		
First	1 982 026 (55.8)	1396 (54.2)
Second	1 569 425 (44.2)	1179 (45.8)
Observation time after dose, d		
Mean (SD)	27.6 (3.7)	27.8 (3.5)
Median (IQR)	30.0 (27.0-30.0)	30.0 (28.0-30.0)
Site of vaccination^b^		
Mass immunization center	29 243 (0.8)	31 (1.2)
Office	1 096 176 (30.9)	871 (33.8)
Outpatient hospital	361 376 (10.2)	324 (12.6)
Pharmacy	1 983 037 (55.8)	1224 (47.5)
State or local public health clinic	67 241 (1.9)	112 (4.3)
Other	14 378 (0.4)	13 (0.5)
Month of vaccination^b^		
December 2020	12 917 (0.4)	12 (0.5)
January 2021	106 041 (3.0)	105 (4.1)
February 2021	216 489 (6.1)	266 (10.3)
March 2021	834 347 (23.5)	849 (33.0)
April 2021	1 248 417 (35.2)	1004 (39.0)
May 2021	758 293 (21.4)	339 (13.2)
June 2021	374 947 (10.6)	0

Abbreviation: SCRI, self-controlled risk interval.

^a^ Patients included in the SCRI analysis had herpes zoster infection in either a risk or control interval. This column includes all vaccine doses from all patients included in the analysis.

^b^ Due to rounding, the percentages for the SCRI population site of vaccination sum to 99.9%, and the percentages for both populations’ month of vaccination sum to greater than 100.0%.

**Figure 2.**
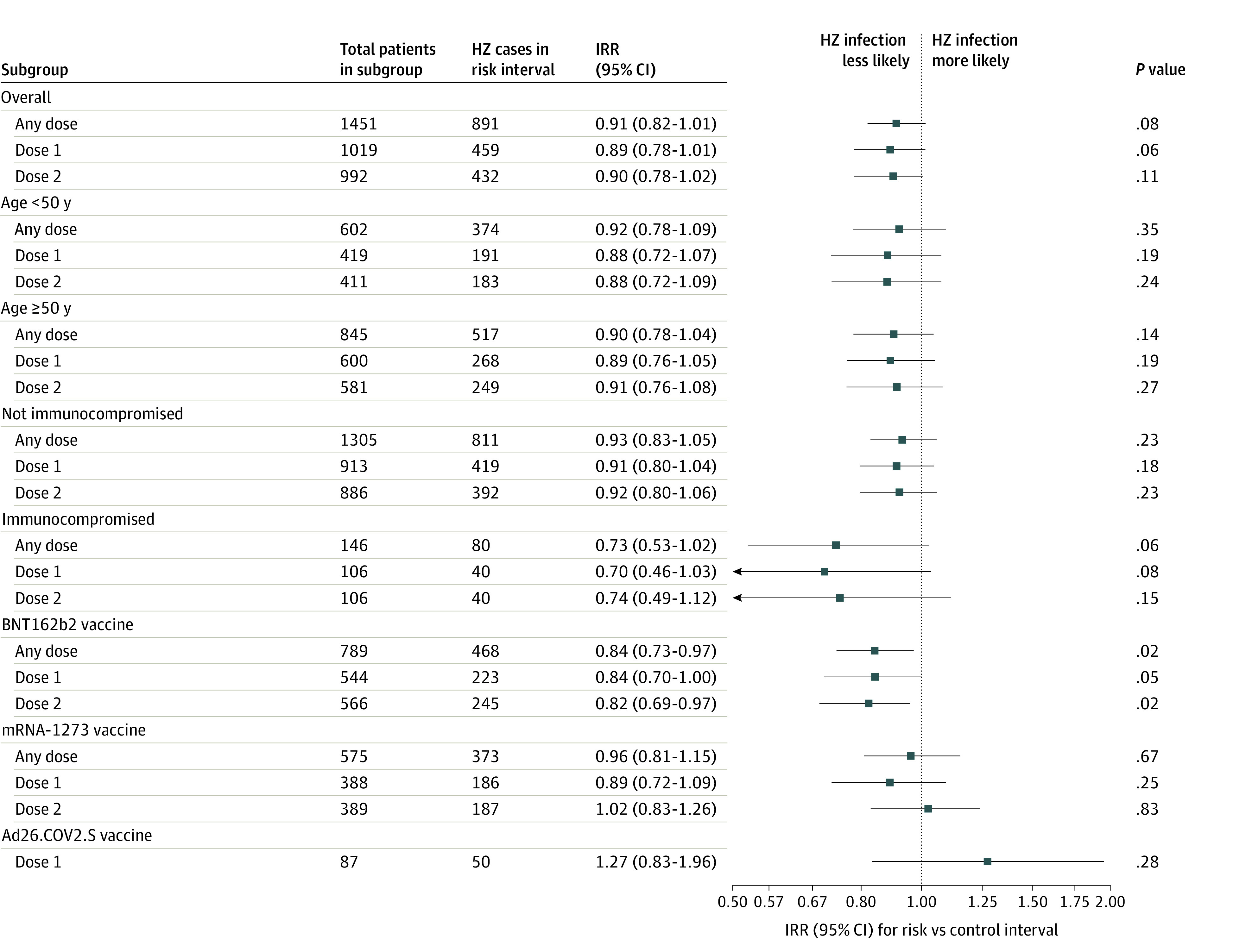
Incidence Rate Ratio of Herpes Zoster After COVID-19 Vaccination With Subgroup Analyses Incidence rate ratios (IRRs), 95% CIs, and *P* values were calculated using conditional Poisson regression models. Age and immunocompromised status were determined at baseline before the first vaccine dose. Immunocompromising conditions included HIV or AIDS, cancer, solid organ transplant, systemic corticosteroid use, and immunosuppressive medication use. Individuals who received the Ad26.COV2.S vaccine, which has a 1-dose regimen, were included in the *any dose* and *dose 1* models but not in the *dose 2* models. Thus, the subgroup results for both models among individuals who received the Ad26.COV2.S vaccine were the same and are only presented once in the figure. HZ indicates herpes zoster. Markers indicate IRRs, and horizontal lines indicate 95% CIs.

There were 891 cases of herpes zoster in the risk interval after COVID-19 vaccination, with 459 cases after the first dose and 432 cases after the second dose. The IRR comparing the risk of herpes zoster during the risk interval after COVID-19 vaccination with the risk of herpes zoster during the control interval was 0.91 (95% CI, 0.82-1.01; *P* = .08) (Figure 2). Assessing each COVID-19 vaccine dose individually, the IRR for herpes zoster after the first dose compared with the control interval was 0.89 (95% CI, 0.78-1.01; *P* = .06), whereas the IRR after the second dose was 0.90 (95% CI, 0.78-1.02; *P* = .11).

Subgroup analyses were conducted by age, immunocompromised status, and type of COVID-19 vaccine. There was no increased risk of herpes zoster after COVID-19 vaccination among individuals aged 50 years and older (IRR, 0.90; 95% CI, 0.78-1.04; *P* = .14) (Figure 2). In addition, immunocompromised status was not associated with an increased risk of herpes zoster after COVID-19 vaccination (IRR, 0.73; 95% CI, 0.53-1.02; *P* = .06). Vaccination with BNT162b2 was associated with a decreased risk of herpes zoster (IRR, 0.84; 95% CI, 0.73-0.97; *P* = .02), and no association with herpes zoster was detected within the mRNA-1273 subgroup (IRR, 0.96; 95% CI, 0.81-1.15; *P* = .67).

Results from the supplemental cohort analysis are shown in the eAppendix and eTables 10 to 13 in the Supplement. In this analysis, the 2 039 854 individuals who received a COVID-19 vaccine (COVID-19 group) were compared with 5 058 394 individuals who received an influenza vaccine in the prepandemic period (prepandemic influenza group) and 4 029 131 individuals who received an influenza vaccine in the early pandemic period (early pandemic influenza group) (eTable 10 in the Supplement). Compared with the prepandemic and early pandemic influenza groups, the COVID-19 group was younger and healthier, and a smaller proportion of individuals in the COVID-19 group vs the influenza groups were of Black race.

In the cohort analysis, after adjustment for demographic characteristics, medical history, and medication use, COVID-19 vaccination was significantly associated with a lower risk of herpes zoster compared with influenza vaccination in the prepandemic period (first dose of COVID-19 vaccine: hazard ratio [HR], 0.78 [95% CI, 0.70-0.86; *P* < .001]; second dose of COVID-19 vaccine: HR, 0.79 [95% CI, 0.71-0.88; *P* < .001]) (eTable 11 in the Supplement). COVID-19 vaccination was not associated with a higher risk of herpes zoster compared with influenza vaccination in the early pandemic period (first dose of COVID-19 vaccine: HR, 0.89 [95% CI, 0.80-1.00; *P* = .05]; second dose: HR, 0.91 [95% CI, 0.81-1.02; *P* = .09]) (eTable 12 in the Supplement).

## Discussion

In this cohort study using data from a large insurance claims database, no increase in the risk of herpes zoster was found after a single dose or full (2-dose) primary series of a COVID-19 vaccine. There was no increase in risk of herpes zoster after COVID-19 vaccination when individuals were stratified by age, immunocompromised status, or type of vaccine administered. These findings from the SCRI analysis were corroborated by the supplementary cohort analysis, in which COVID-19 vaccination was not found to be associated with a higher risk of herpes zoster compared with influenza vaccination in either the prepandemic or early pandemic periods.

Numerous conditions, such as Bell palsy, myocarditis, and thromboembolic events, have been studied as potential adverse events after COVID-19 vaccination.^3^ Herpes zoster infection after COVID-19 vaccination has been repeatedly reported in the literature.^6,7,8,20,21^ Analyses of vaccine adverse event reporting systems have also revealed an increase in reporting of herpes zoster.^22,23^ Several mechanisms have been suggested by which latent varicella zoster virus could be reactivated after vaccination. Vaccine-related immunomodulation has been thought to explain cases of herpes zoster reactivation associated with inactivated influenza, hepatitis A, and rabies virus with Japanese encephalitis vaccines,^24^ although no epidemiological assessments of the association between herpes zoster and any of those vaccines have been conducted. Furthermore, mRNA vaccines stimulate toll-like receptor signaling, a pathway involved in the latency and reactivation of varicella zoster virus, so there is a plausible mechanism by which the mRNA-based COVID-19 vaccines could lead to herpes zoster infection.^25,26,27^

Cohort studies have generated mixed data regarding the risk of herpes zoster infection after COVID-19 vaccination. Among the limited existing studies, 1 claims-based analysis^28^ and 2 Israeli cohort studies^29,30^ found no increased risk, whereas 2 other studies^4,31^ identified an increased risk of herpes zoster infection after COVID-19 vaccination. Compared with previous work,^4,28,29,30,31^ our study had a larger sample, which enabled us to control for confounders that were not included in other studies, such as measures of health care use, history of zoster vaccination, and comorbidity-related risk factors for herpes zoster rather than weighted indices. In addition, our study included a racially and ethnically heterogenous cohort compared with the studies conducted in Israel^29,30^ and may therefore better represent postvaccination herpes zoster event risk in diverse populations. In a study of herpes zoster–associated hospitalization after receipt of the BNT162b2 and CoronaVac (Sinovac Life Sciences Company) vaccines, an increased risk after vaccination was identified.^32^ It is possible that cases of herpes zoster after vaccination differ in disease course; however, further research is needed to assess this possibility.

### Strengths and Limitations

This study has several strengths. The study adds to the body of literature examining the safety profile of COVID-19 vaccines in clinical practice. Our study is strengthened by its large sample, which allowed us to evaluate a relatively rare but clinically important outcome. Our study is also novel in that it modeled the risk of herpes zoster infection separately after dose 1 and dose 2 for multidose COVID-19 vaccines. This distinction provides clinically meaningful information given that the rate of adverse events is known to be different after each dose.^33^ We modeled the risk of herpes zoster using 2 different study designs and analyses, including adjustment for a variety of confounders, which added to the rigor of this study. The use of the SCRI design allowed us to control for time-invariant factors, eliminating many potential sources of confounding that occur in cohort studies. In addition, by using an SCRI design and including historical comparison groups who received influenza vaccines in the cohort design, we were able to avoid misclassification of exposure that can occur when comparing vaccinated individuals with unvaccinated individuals in the same period. Comparisons with CDC COVID-19 vaccination data^34^ suggest that the OLDW best captured vaccines administered in traditional health care settings after February 2021. Therefore, we could not be certain that an individual without a record of COVID-19 vaccination in the database did not receive a vaccine at a site outside of their usual health care setting. The methods used in our study allowed us to avoid this established limitation of studying COVID-19 vaccine–related events using data from a claims database.

This study also has limitations. Due to both the limited granularity of insurance claims data and the change in health care use over the course of the pandemic, outcome misclassification is possible. To overcome potential outcome misclassification, we used a strict definition of herpes zoster that required a prescription for a new antiviral medication or a dose increase in antiviral medication within 5 days of diagnosis. However, individuals experiencing herpes zoster during the pandemic may have avoided or delayed seeking care, and the likelihood of seeking care may have varied over time depending on the COVID-19 case rate or a patient’s time since vaccination. Another limitation is that the OLDW does not include individuals with basic Medicare or Medicaid plans or those who are uninsured; thus, the sample included in our study could represent a more economically advantaged population. Based on the comparison of baseline characteristics between the groups in the supplemental cohort analysis, some demographic groups, such as Black individuals, were underrepresented in the COVID-19–vaccinated group, and those who received a COVID-19 vaccine were younger and healthier overall than those who received an influenza vaccine. Because those included in the SCRI analysis are a subset of the COVID-19–vaccinated cohort who experienced herpes zoster during the study period, our results may not be generalizable to the full population of individuals who received a COVID-19 vaccine in the US.

## Conclusions

This cohort study using data from a large insurance claims database did not find an association between COVID-19 vaccination and an increased risk of herpes zoster infection. These results may help to address concerns about the safety profile of the COVID-19 vaccines among patients and clinicians. Further research is needed to assess the implications of additional COVID-19 vaccine doses for the risk of herpes zoster infection.

## Notes

Supplement. **eAppendix.** eMethods and eResults **eFigure.** Study Design and Population Flow Diagram **eTable 1.** Procedure and Drug Codes for COVID-19 Vaccines From December 11, 2020, Through June 30, 2021 **eTable 2.** Corticosteroids Used to Identify Herpes Zoster Outcome **eTable 3.** Antiviral Medications and Dose Levels Used to Identify Herpes Zoster Outcome and Covariate Status **eTable 4.** Procedure Codes for Influenza Vaccines From January 1, 2018, Through July 31, 2021 **eTable 5.** *ICD-10* Codes Used to Identify Comorbidity Status **eTable 6.** Search Terms Used to Identify Herpes Zoster Vaccination Status **eTable 7.** Systemic Corticosteroids and Other Immunosuppressants Used to Determine Covariate Status **eTable 8.** *ICD-10* Codes and Descriptions Used to Identify COVID-19 Infection **eTable 9.** Negative Control Outcomes and Corresponding *ICD-10* Codes **eTable 10.** Characteristics of Cohort Study Population by Vaccine Exposure **eTable 11.** Risk of Herpes Zoster Infection Among COVID-19 Vaccinated vs Influenza Vaccinated, Prepandemic **eTable 12.** Risk of Herpes Zoster Infection Among COVID-19 Vaccinated vs Influenza Vaccinated, Early Pandemic **eTable 13.** Comparison of Adjusted Hazard Ratios of Vaccination Type (COVID-19 vs Prepandemic Influenza and COVID-19 vs Early Pandemic Influenza) for Herpes Zoster and 3 Negative Control Outcomes
eReferences
