# Supplementary material for: Assessment of Herpes Zoster Risk Among Recipients of COVID-19 Vaccine
Source: JAMA Netw Open. 2022 Nov 16;5(11):e2242240. doi: 10.1001/jamanetworkopen.2022.42240 (PMC9669817; doi:10.1001/jamanetworkopen.2022.42240)
Supplement: Supplement. — eAppendix. eMethods and eResults eFigure. Study Design and Population Flow Diagram eTable 1. Procedure and Drug Codes for COVID-19 Vaccines From December 11, 2020, Through June 30, 2021 eTable 2. Corticosteroids Used to Identify Herpes Zoster Outcome eTable 3. Antiviral Medications and Dose Levels Used to Identify Herpes Zoster Outcome and Covariate Status eTable 4. Procedure Codes for Influenza Vaccines From January 1, 2018, Through July 31, 2021 eTable 5. ICD-10 Codes Used to Identify Comorbidity Status eTable 6. Search Terms Used to Identify Herpes Zoster Vaccination Status eTable 7. Systemic Corticosteroids and Other Immunosuppressants Used to Determine Covariate Status eTable 8. ICD-10 Codes and Descriptions Used to Identify COVID-19 Infection eTable 9. Negative Control Outcomes and Corresponding ICD-10 Codes eTable 10. Characteristics of Cohort Study Population by Vaccine Exposure eTable 11. Risk of Herpes Zoster Infection Among COVID-19 Vaccinated vs Influenza Vaccinated, Prepandemic eTable 12. Risk of Herpes Zoster Infection Among COVID-19 Vaccinated vs Influenza Vaccinated, Early Pandemic eTable 13. Comparison of Adjusted Hazard Ratios of Vaccination Type (COVID-19 vs Prepandemic Influenza and COVID-19 vs Early Pandemic Influenza) for Herpes Zoster and 3 Negative Control Outcomes eReferences [file jamanetwopen-e2242240-s001.pdf]

Supplemental Online Content

Akpandak I, Miller DC, Sun Y, Arnold BF, Kelly JD, Acharya NR. Assessment of herpes zoster risk among recipients of COVID-19 vaccine. *JAMA Netw Open*. 2022;5(11):e2242240. doi:10.1001/jamanetworkopen.2022.42240

eAppendix. eMethods and eResults

eFigure. Study Design and Population Flow Diagram

eTable 1. Procedure and Drug Codes for COVID-19 Vaccines From December 11, 2020, Through June 30, 2021

eTable 2. Corticosteroids Used to Identify Herpes Zoster Outcome

eTable 3. Antiviral Medications and Dose Levels Used to Identify Herpes Zoster Outcome and Covariate Status

eTable 4. Procedure Codes for Influenza Vaccines From January 1, 2018, Through July 31, 2021

eTable 5. *ICD-10* Codes Used to Identify Comorbidity Status

eTable 6. Search Terms Used to Identify Herpes Zoster Vaccination Status

eTable 7. Systemic Corticosteroids and Other Immunosuppressants Used to Determine Covariate Status

eTable 8. *ICD-10* Codes and Descriptions Used to Identify COVID-19 Infection

eTable 9. Negative Control Outcomes and Corresponding *ICD-10* Codes

eTable 10. Characteristics of Cohort Study Population by Vaccine Exposure

eTable 11. Risk of Herpes Zoster Infection Among COVID-19 Vaccinated vs Influenza Vaccinated, Prepandemic

eTable 12. Risk of Herpes Zoster Infection Among COVID-19 Vaccinated vs Influenza Vaccinated, Early Pandemic

eTable 13. Comparison of Adjusted Hazard Ratios of Vaccination Type (COVID-19 vs Prepandemic Influenza and COVID-19 vs Early Pandemic Influenza) for Herpes Zoster and 3 Negative Control Outcomes

eReferences

This supplemental material has been provided by the authors to give readers additional information about their work.

© 2022 Akpandak I et al. *JAMA Network Open*.

eAppendix. eMethods and eResults

1.1 eMethods.

Supplementary cohort analysis

As a supplementary analysis, a cohort study design was implemented. This study following the Strengthening the Reporting of Observational Studies in Epidemiology (STROBE) reporting guideline for cohort studies. The COVID-19 vaccinated cohort consisted of COVID-19 vaccinated individuals who met the inclusion criteria outlined in the main text. Refer to the eFigure for a study flow diagram demonstrating the inclusion criteria for each study population. Due to incomplete capture of COVID-19 vaccination records in US-based claims data,[1] we compared the COVID-19 vaccinated cohort to two historical influenza vaccinated cohorts. The pre-pandemic influenza vaccinated cohort was comprised of individuals who received an influenza vaccine from January 1, 2018 to December 31, 2019. The early pandemic influenza-vaccinated cohort was comprised of individuals who received an influenza vaccine from March 1, 2020 to November 30, 2020. Influenza-vaccinated patients were required to be continuously enrolled in both medical and pharmacy coverage from 270 days prior to the date of first recorded influenza vaccination (index date) through 30 days following the index date. Influenza vaccines were identified similarly to COVID-19 vaccines (eTable 4). Patients with a previous diagnosis of herpes zoster (HZ) in the 270 days prior to the index date were excluded. Patients who received both an influenza vaccine and a COVID-19 vaccine within the same 30-day period were excluded from the study. Patients could be included in the COVID-19 vaccinated group, and either or both influenza-vaccinated groups if they met the inclusion criteria for each group.

Demographic characteristics including age, gender, and race/ethnicity were assessed on the index date. Race/ethnicity is assigned by an external vendor who uses a rule-based system that combines analysis of first names, middle names, surnames, and surname prefixes and suffixes with geographic criteria. Optum Labs then assigns these ethnicity values into one of five compliance-determined race/ethnicity code values: W (Non-Hispanic White), B (Non-Hispanic Black), H (Hispanic), A (Asian), and U (Unknown). Baseline comorbidities were assessed in the 270 days prior to the index date using ICD-10 codes (eTable 5). Ambulatory healthcare utilization (outpatient and doctor's office encounters) was summarized as the visit count within the 270 days prior to the index date, and inpatient healthcare utilization (inpatient, emergency room, or long-term care encounters) was summarized as a binary variable indicating whether the patient had any of these encounter types in the 270 days before the index date. In addition to comorbidities and healthcare utilization, receipt of recombinant zoster vaccination (RZV; Shingrix, GlaxoSmithKline) or zoster live vaccine (ZVL; Zostavax, Merck) in the five years prior to the index date was also captured (eTable 6). Systemic corticosteroid use, other immunosuppressant use, and systemic antiviral medication use was assessed at the time of vaccination (eTable 3, 7). In the COVID-19 vaccinated and early pandemic influenza-vaccinated groups, recent COVID-19 infection was assessed in the 30 days prior to the start of the risk interval up to the event/censor date (eTable 8). Incident HZ following COVID-19 vaccination was assessed as described in the main text. Influenza-vaccinated individuals were evaluated for HZ in the 30 days after vaccination.

Cox proportional hazards models were used to calculate the unadjusted and adjusted hazards of HZ among COVID-19 vaccinated individuals compared to influenza vaccinated individuals, with separate models used to compare COVID-19 vaccinated patients to the two influenza vaccinated groups. Adjusted models included age, gender, race/ethnicity, baseline comorbidities, healthcare utilization, history of zoster vaccination, systemic corticosteroid use, other immunosuppressant use, systemic antiviral use, and recent COVID-19 infection when applicable. Individuals were observed up to the first occurrence of HZ, so risk periods following subsequent COVID-19 vaccine doses were not included in the analysis if the individual experienced HZ after the first dose. Robust standard errors clustered on individuals were used to account for administration of multiple doses of COVID-19 vaccine, or receipt of both an influenza vaccine and COVID-19 vaccine.

Because there may be inherent differences between those who received influenza vaccines in years past and those who received a COVID-19 vaccine in 2021, we conducted a bias assessment to detect potential residual unmeasured confounding using three negative control outcomes occurring during the periods after COVID-19 vaccination and influenza vaccination. The negative control outcomes selected were kidney or urethral stone, pyelonephritis, and irritant contact dermatitis. The outcomes chosen were acute conditions with shared confounders with HZ and were expected to have no association with COVID-19 vaccination or influenza vaccination (see eTable

© 2022 Akpandak I et al. *JAMA Network Open*.

9 for ICD-10 codes). Thus, an observed association between vaccine type and any of the control outcomes could indicate that hazard ratios for HZ were biased.

Antiviral dose calculation and escalation for identifying HZ cases

Antiviral doses were evaluated by calculating the average daily dose in milligrams (mg/day) based on the unit quantity dispensed, days' supply, and dosage per unit. Average mg/day and the drug name was used to determine baseline dose level before the HZ diagnosis and in the five days after an HZ diagnosis based on the levels specified in eTable 3. The levels specified were based on levels defined in previous literature and confirmed by Dr. Nisha Acharya.[2] A dose increase was defined by an increase in the dose level, rather than simply an increase in the mg/day, as different medications have different standard daily dose ranges, and the average daily dose calculated from pharmacy claims may not correspond exactly to a standard daily dose.

1.2 eResults.

There were 2686 cases of HZ following pre-pandemic influenza vaccinations and 1899 cases of HZ following early pandemic influenza vaccinations, corresponding to an event rate of 6.5 per 1 000 person-years and 5.7 per 1000 person-years, respectively. There were 459 HZ cases following the first dose of COVID-19 vaccine and 432 HZ cases following the second dose of COVID-19 vaccine, corresponding to an event rate of 3.3 per 1 000 person-years and 3.4 per 1000 person-years, respectively. The median time to HZ following COVID-19 vaccine was 13 days (IQR: 7 - 20) after dose 1 and 16 days (IQR: 8 - 23) after dose 2. The median time to HZ following influenza vaccine was 15 days (IQR: 8 - 23) in both the pre-pandemic and early pandemic period.

After adjustment for demographic characteristics, medical history, and medication use, COVID-19 vaccination was significantly associated with a decreased risk of HZ compared to the pre-pandemic influenza vaccinated cohort (1st dose: HR = 0.78, 95% CI: 0.70 – 0.86, $p < 0.001$; 2nd dose: HR = 0.79, 95% CI: 0.71 – 0.88, $p < 0.001$; eTable 11). COVID-19 vaccination was not associated with an increased risk of HZ compared to the early pandemic influenza vaccinated cohort (1st dose: HR = 0.89, 95% CI: 0.80 – 1.00, p = 0.05; 2nd dose: HR = 0.91, 95% CI: 0.81 – 1.02, p = 0.09; eTable 12). In the analysis of negative control outcomes selected to address potential residual confounding after covariate adjustment, COVID-19 vaccination was observed to have a small protective or null effect on the chosen outcomes compared to influenza vaccination in the pre-pandemic and early pandemic periods (eTable 13).

© 2022 Akpandak I et al. *JAMA Network Open*.

eFigure. Study Design and Population Flow Diagram

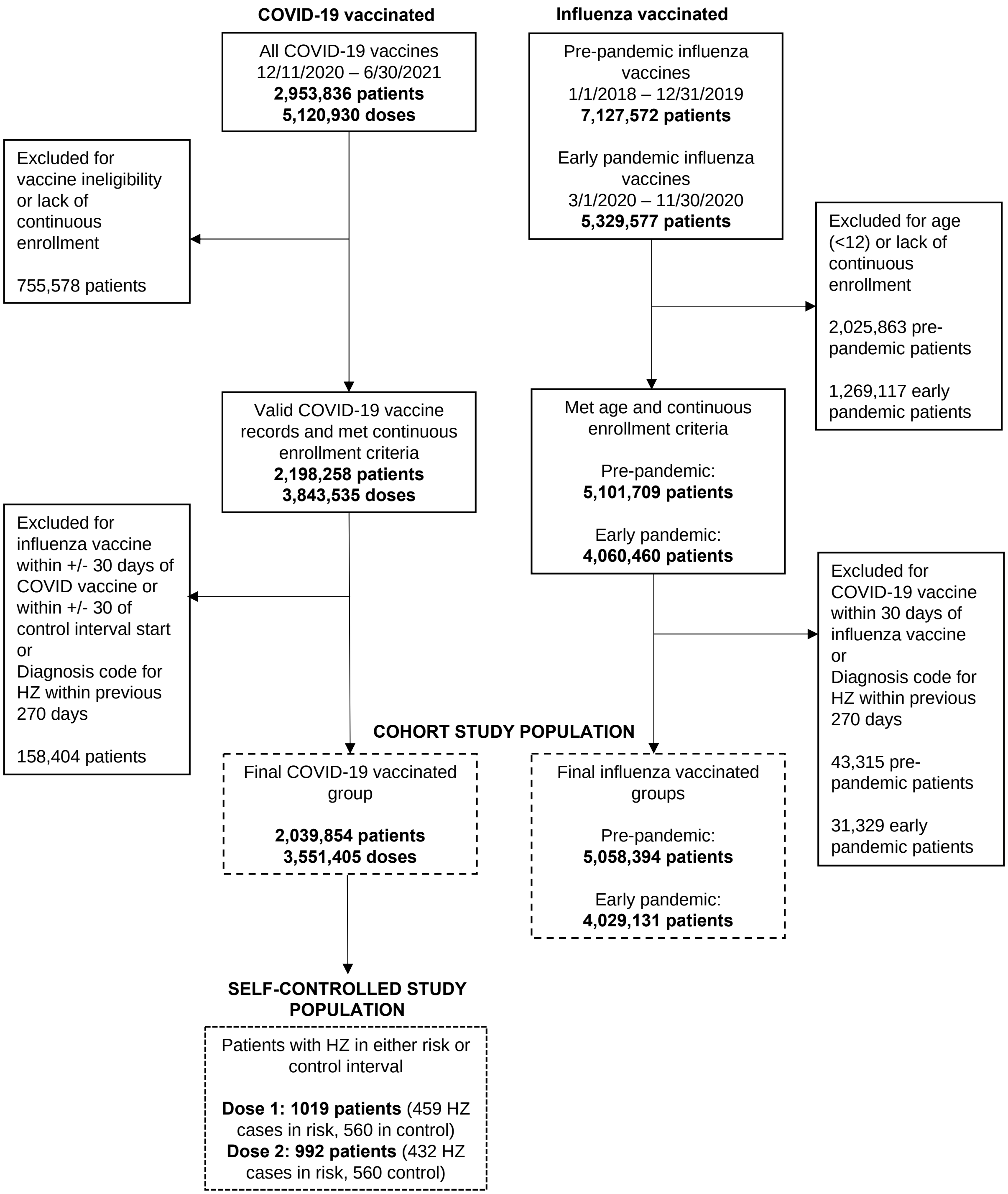

© 2022 Akpandak I et al. *JAMA Network Open*.

eTable 1. Procedure and Drug Codes for COVID-19 Vaccines From December 11, 2020, Through June 30, 2021

**Characteristic**	**BNT-162b2 (Pfizer)**	**mRNA-1273 (Moderna)**	**Ad26.COV2.S (Janssen)**
Emergency use authorization date (ages authorized)	12/11/2020 (≥16)<br>5/10/2021 (12-15)	12/18/2020 (≥18)	2/27/2021 (≥18)
Doses	2	2	1
Vaccine product procedure codes	91300	91301	91303
Immunization administration procedure codes	0001A (1st dose)<br>0002A (2nd dose)	0011A (1st dose)<br>0012A (2nd dose)	0031A
NDC 11 carton codes (multi-dose)	59267-1000-02<br>59267-1000-03	80777-0273-98<br>80777-0273-99	59676-0580-15
NDC 11 vial codes (multi-dose)	59267-1000-01	80777-0273-15<br>80777-0273-10	59676-0580-05

In addition to NDC codes, we used a text search of '%covid%vac%' as the generic or brand name in the pharmacy claims, where '%' indicates a wild card (i.e. any characters allowed in those spaces).

© 2022 Akpandak I et al. *JAMA Network Open*.

eTable 2. Corticosteroids Used to Identify Herpes Zoster Outcome

Corticosteroid type	Medication name
**Ophthalmic corticosteroids**	Blephamide<br>Budesonide<br>Cortisone acetate<br>Dexamethasone<br>Difluprednate<br>Fluorometholone acetate<br>Loteprednol etabonate<br>Medrysone<br>Methylprednisolone<br>Prednisolone<br>Prednisone<br>Rimexolone<br>Triamcinolone
**Oral corticosteroids**	Betamethasone<br>Budesonide<br>Cortisone acetate<br>Deflazacort<br>Dexamethasone<br>Hydrocortisone<br>Methylprednisolone<br>Prednisolone<br>Prednisone<br>Triamcinolone

© 2022 Akpandak I et al. *JAMA Network Open*.

eTable 3. Antiviral Medications and Dose Levels Used to Identify Herpes Zoster Outcome and Covariate Status

Dose level	Antiviral generic name (brand name) & dose range in mg/day		
	**ACYCLOVIR (ZOVIRAX)**	**FAMCICLOVIR (FAMVIR)**	**VALACYCLOVIR (VALTREX)**
0 - very low	0 < dose < 400	0 < dose < 125	0 < dose < 250
1 - low	400 ≤ dose < 1200	--	250 ≤ dose < 750
2 - medium low	1200 ≤ dose < 2000	125 ≤ dose < 375	750 ≤ dose < 1250
3 - medium	2000 ≤ dose < 2800	375 ≤ dose < 750	1250 ≤ dose < 1750
4 - medium high	2800 ≤ dose < 3600	750 ≤ dose < 1250	1750 ≤ dose < 2500
5 - high	dose ≥ 3600	dose ≥ 1250	dose ≥ 2500

Doses are in mg.

© 2022 Akpandak I et al. *JAMA Network Open*.

eTable 4. Procedure Codes for Influenza Vaccines From January 1, 2018, Through July 31, 2021

Administration & Diagnosis Codes	Vaccine Codes & Descriptors
90630	Influenza virus vaccine, quadrivalent (IIV4), split virus, preservative free, for intradermal use
90653	Influenza vaccine, inactivated (IIV), subunit, adjuvanted, for intramuscular use
90654	Influenza virus vaccine, trivalent (IIV3), split virus, preservative-free, for intradermal use
90655	Influenza virus vaccine, trivalent (IIV3), split virus, preservative free, 0.25 mL dosage, for intramuscular use
90656	Influenza virus vaccine, trivalent (IIV3), split virus, preservative free, 0.5 mL dosage, for intramuscular use
90657	Influenza virus vaccine, trivalent (IIV3), split virus, 0.25 mL dosage, for intramuscular use
90658	Influenza virus vaccine, trivalent (IIV3), split virus, 0.5 mL dosage, for intramuscular use
90660	Influenza virus vaccine, trivalent, live (LAIV3), for intranasal use
90662	Influenza virus vaccine (IIV), split virus, preservative free, enhanced immunogenicity via increased antigen content, for intramuscular use
90672	Influenza virus vaccine, quadrivalent, live (LAIV4), for intranasal use
90673	Influenza virus vaccine, trivalent (RIV3), derived from recombinant DNA, hemagglutinin (HA) protein only, preservative and antibiotic free, for intramuscular use
90674	Influenza virus vaccine, quadrivalent (ccIIV4), derived from cell cultures, subunit, preservative and antibiotic free, 0.5 mL dosage, for intramuscular use
90682	Influenza virus vaccine, quadrivalent (RIV4), derived from recombinant DNA, hemagglutinin (HA) protein only, preservative and antibiotic free, for intramuscular use
90685	Influenza virus vaccine, quadrivalent (IIV4), split virus, preservative free, 0.25 mL dosage, for intramuscular use
90686	Influenza virus vaccine, quadrivalent (IIV4), split virus, preservative free, 0.5 mL dosage, for intramuscular use
90687	Influenza virus vaccine, quadrivalent (IIV4), split virus, 0.25 mL dosage, for intramuscular use
90688	Influenza virus vaccine, quadrivalent (IIV4), split virus, 0.5 mL dosage, for intramuscular use
90689	Influenza virus vaccine quadrivalent (IIV4), inactivated, adjuvanted, preservative free, 0.25mL dosage, for intramuscular use
90694	Influenza virus vaccine, quadrivalent (aIIV4), inactivated, adjuvanted, preservative free, 0.5 mL dosage, for intramuscular use
90756	Influenza virus vaccine, quadrivalent (ccIIV4), derived from cell cultures, subunit, antibiotic free, 0.5 mL dosage, for intramuscular use
Q2034	Influenza virus vaccine, split virus, for intramuscular use (agriflu)
Q2035	Influenza virus vaccine, split virus, when administered to individuals 3 years of age and older, for intramuscular use (afluria)
Q2036	Influenza virus vaccine, split virus, when administered to individuals 3 years of age and older, for intramuscular use (flulaval)
Q2037	Influenza virus vaccine, split virus, when administered to individuals 3 years of age and older, for intramuscular use (fluvirin)

© 2022 Akpandak I et al. *JAMA Network Open*.

Administration & Diagnosis Codes	Vaccine Codes & Descriptors
Q2038	Influenza virus vaccine, split virus, when administered to individuals 3 years of age and older, for intramuscular use (fluzone)
Q2039	Influenza virus vaccine, not otherwise specified
G0008	Administration of influenza virus vaccine

In addition to procedure codes, we used a text search of '%flu%vac%' as the generic or brand name in the pharmacy claims, where '%' indicates a wild card (i.e. any characters allowed in those spaces).

© 2022 Akpandak I et al. *JAMA Network Open*.

eTable 5. *ICD-10* Codes Used to Identify Comorbidity Status

Condition	ICD-10 codes
**Asthma**	J45.x, J46.x
**Autoimmune disease**	M02.9, (reactive arthritis)<br>M05.x, M06.x, (rheumatoid arthritis)<br>M07.x, (psoriatic and enteropathic arthritis)<br>M08.xx, M09.x, (juvenile arthritis)<br>M10.x, (gout)<br>M30.x, (polyarteritis nodosa)<br>M31.3, (granulomatosis with polyangiitis)<br>M31.5, M31.6, M35.3, (polymyalgia rheumatica)<br>M32.1, M32.8, M32.9, L93.x (systemic lupus erythematosus)<br>M33.x, (dermatopolymyositis)<br>M34.x, (systemic sclerosis)<br>M35.0, (Sjogren's)<br>M35.2, (Behcet's)<br>M45.x, (ankylosing spondylitis)<br>M47.819, (Undifferentiated spondyloarthropathy)<br>M94.1 (relapsing polychondritis)<br>G35.x, (multiple sclerosis)<br>G61.0, (Guillain-Barré)<br>G70.0, (myasthenia gravis)<br>D59.1, (autoimmune hemolytic anemia)<br>D86.x, (sarcoidosis)<br>K50.x, K51.x, (Crohn's and ulcerative colitis)<br>K75.4, (autoimmune hepatitis)<br>L80.x, (vitiligo)<br>L63.x, (alopecia areata)<br>L40.x, (psoriasis vulgaris)<br>L10.x, L12.x, (pemphigus and pemphigoid)<br>K90.0, (celiac disease)<br>E06.3, (autoimmune thyroiditis)<br>D69.3, (Immune thrombocytopenic purpura)<br>E27.1, (primary adrenocortical insufficiency)<br>E31.0, (autoimmune polyglandular failure)<br>K74.3, (primary biliary cirrhosis)<br>N05.8, N05.9 (Tubulointerstitial nephritis)<br>H20.9, H20.04x, H20.1x, H20.02x, H20.00, H20.01x, H30.2x, H35.06x, H20.82x, H30.81x, H44.11x, H44.13x, H30.01x, H30.02x, H30.03x, H30.04x, H30.14x, H30.89x,<br>H30.9x, H31.22, H31.8 (non-infectious uveitis)
**Cancer**	C00.x-C26.x, C30.x-C34.x, C37.x-C41.x, C43.x, C45.x-C58.x, C60.x-C76.x, C81.x-C85.x, C88.x, C90.x-C97.x (malignancy)<br><br>C77.x-C80.x (metastatic solid tumor)
**Cardiovascular diseases**	109.81 (rheumatic heart failure)<br>I11.0, I13.0, I13.2, (hypertensive heart disease with heart failure)<br>I21.x, I22.x, I23.x, I25.x, (ischemic – excluding angina and other acute ischemic heart disease)<br>I42.x, I43.x, (cardiomyopathy)<br>I50.x (heart failure)
**Chronic kidney diseases**	I12.xx, I13.xx, (hypertensive renal disease)

© 2022 Akpandak I et al. *JAMA Network Open*.

Condition	ICD-10 codes
	N03.2-N03.7, (chronic nephritic syndrome)<br>N05.2-N05.7, (unspecified nephrotic syndrome)<br>N18.xx, N19.x, (chronic kidney disease and failure)<br>N25.0, (impaired renal tubular function)<br>Z49.xx, (dialysis)<br>Z99.2 (dependence on renal dialysis)<br>Q61.x (cystic kidney disease),<br>E10.2, E11.2, E12.2, E13.2, E14.2 (DM with renal complications)
**Chronic lung diseases**	I27.8, I27.9, (pulmonary heart disease)<br>J40.x, J41.x, J42.x, (chronic bronchitis)<br>J43.x, J44.x, (COPD)<br>J47.x, (bronchiectasis)<br>J60.x, J61.x, J62.x, J63.x, J64.x, J65.x, J66.x, J67.x, (pneumoconiosis/pneumonitis)<br>J68.4, J70.1, J70.3 (chronic respiratory conditions due to external agents)<br>J84.x (interstitial pulmonary diseases)<br>E84.0 (cystic fibrosis with pulmonary manifestations)
**Diabetes mellitus**	E10.xx (type 1), E11.xx (type 2), E12.xx (malnutrition DM), E13.xx (other), E14.xx (unspecified), O24.xx (gestational)
**HIV/AIDS**	B20.x, B97.35, R75.x, Z21.x
**Solid organ transplantation**	Z94.xx

Abbreviations: HIV=human immunodeficiency virus; AIDS=acquired immunodeficiency syndrome.

© 2022 Akpandak I et al. *JAMA Network Open*.

eTable 6. Search Terms Used to Identify Herpes Zoster Vaccination Status

Zoster vaccine type	Brand name	CPT code
Zoster vaccine live	Zostavax	90736
Recombinant zoster vaccine	Shingrix	90750

© 2022 Akpandak I et al. *JAMA Network Open*.

eTable 7. Systemic Corticosteroids and Other Immunosuppressants Used to Determine Covariate Status

Medication type	Medication name	
**Systemic corticosteroids**<br>Routes: oral, intravenous	BAYCADRON<br>BETAMETHASONE<br>BUDESONIDE<br>CORTEF<br>CORTISONE ACETATE<br>DECADRON<br>DECAMETH<br>DECAREX<br>DEFLAZACORT<br>DELTASONE<br>DEXAMETHASONE<br>DEXAPACK<br>DEXONE<br>DEXPAK<br>EMFLAZA<br>ENTOCORT<br>HYDROCORTISONE<br>LISACORT<br>MEDROL	METHYLPRED<br>METHYLPREDNISOLONE<br>METICORTEN<br>MILLIPRED<br>ORAPRED<br>ORTIKOS<br>PREDNISOLONE<br>PREDNISONE<br>PRELONE<br>RAYOS<br>RAYOS<br>TAPERDEX<br>TRIAMCINOLONE<br>UCERIS<br>ULACORT<br>ZCORT<br>ZODEX
**Other immunosuppressants**<br>Routes: oral, intravenous, injection, or subcutaneous	**I. Antineoplastics**<br>ABARELIX<br>ABRAXANE<br>ACTIMMUNE<br>ADRIAMYCIN<br>ADRUCIL<br>AFINITOR<br>ALDESLEUKIN<br>ALEMTUZUMAB<br>ALFERON<br>ALIMTA<br>ALITRETINOIN<br>ALKERAN<br>ALTRETAMINE<br>AMINOGLUTETHIMIDE<br>AMINOLEVULINIC<br>ANASTROZOLE<br>ARIMIDEX<br>AROMASIN<br>ARRANON<br>ARSENIC TRIOXIDE<br>ASPARAGINASE<br>AVASTIN<br>AXITINIB<br>AZACITIDINE<br>BCG<br>BENDAMUSTINE<br>BEVACIZUMAB<br>BEXAROTENE<br>BEXXAR<br>BICALUTAMIDE<br>BICNU	SILTUXIMAB<br>SOLTAMOX<br>SORAFENIB<br>SPRYCEL<br>STIVARGA<br>STREPTOZOCIN<br>SUNITINIB<br>SYLVANT<br>TRIPTORELIN<br>TRASTUZUMAB<br>SUTENT<br>TABLOID<br>TAMOXIFEN<br>TARABINE<br>TARCEVA<br>TARGRETIN<br>TASIGNA<br>TAXOTERE<br>TEMODAR<br>TEMOZOLOMIDE<br>TEMSIROLIMUS<br>TENIPOSIDE<br>TESLAC<br>TESTOLACTONE<br>THERACYS<br>THIOGUANINE<br>THIOPLEX<br>THIOTEPA<br>TICE BCG<br>TIOGUANINE<br>TOPOSAR<br>TOPOTECAN

© 2022 Akpandak I et al. *JAMA Network Open*.

Medication type	Medication name	
	BLENOXANE	TOREMIFENE
	BLEOMYCIN	TORISEL
	BORTEZOMIB	TOSITUMOMAB
	BOSULIF	TRABECTEDIN
	BOSUTINIB	TRAMETINIB
	BUSULFAN	TREANDA
	BUSULFEX	TRELSTAR
	CABOMETYX	TREXALL
	CABOZANTINIB	TRIFLURIDINE/TIPIRACIL
	CAMPATH	TRISENOX
	CAMPTOSAR	TYKERB
	CAPECITABINE	UPLIZNA
	CAPRELSA	UVADEX
	CARBOPLATIN	VALRUBICIN
	CARFILZOMIB	VALSTAR
	CARMUSTINE	VANDETANIB
	CASODEX	VECTIBIX
	CEENU	VELCADE
	CERUBIDINE	VEMURAFENIB
	CETUXIMAB	VEPESID
	CHLORAMBUCIL	VESANOID
	CHLORMETHINE	VIADUR
	CISPLATIN	VIDAZA
	CLADRIBINE	VINBLASTINE
	CLOFARABINE	VINCASAR
	CLOLAR	VINCRISTINE
	COMETRIQ	VINORELBINE
	COSMEGEN	VISMODEGIB
	CRIZOTINIB	VORINOSTAT
	CYCLOPHOSPHAMIDE	VOTRIENT
	CYTADREN	VUMON
	CYTARABINE	XALKORI
	CYTOSAR	XELODA
	CYTOXAN	YONDELIS
	DACARBAZINE	ZALTRAP
	DACOGEN	ZANOSAR
	DACTINOMYCIN	ZEVALIN
	DASATINIB	ZOLADEX
	DAUNORUBICIN	ZOLINZA
	DAUNOXOME	ZORTRESS
	DECITABINE	
	DEGARELIX	**II. Antiarthritics**
	DENILEUKIN DIFTITOX	CERTOLIZUMAB
	DOCETAXEL	CIMZIA
	DOXIL	PENICILLAMINE
	DOXORUBICIN	ANAKINRA
	DROXIA	KINERET
	DTIC	ADALIMUMAB
	EFUDEX	ENBREL
	ELIGARD	ETANERCEPT
	ELLENCE	HUMIRA
	ELOXATIN	LEFLUNOMIDE
	ELSPAR	ARAVA
	EMCYT	AURANOFIN

© 2022 Akpandak I et al. *JAMA Network Open*.

Medication type	Medication name	
	EPIRUBICIN	AUROTHIOGLUCOSE
	ERBITUX	THIOMALATE
	ERLOTINIB	ABATACEPT
	ESTRAMUSTINE	ORENCIA
	ETOPOPHOS	REMICADE
	ETOPOSIDE	RHEUMATREX
	EVEROLIMUS	SULFASALAZINE
	EXEMESTANE	AZULFIDINE
	FARESTON	INFLIXIMAB
	FASLODEX	TOCILIZUMAB
	FEMARA	ACTEMRA
	FLOXURIDINE	SARILUMAB
	FLUDARA	KEVZARA
	FLUDARABINE	IXEKIZUMAB
	FLUOROPLEX	TALTZ
	FLUOROURACIL	SECUKINUMAB
	FLUTAMIDE	COSENTYX
	FOLOTYN	USTEKINUMAB
	FUDR	STELARA
	FULVESTRANT	RISANKIZUMAB
	GEFITINIB	SKYRIZI
	GEMCITABINE	CANAKINUMAB
	GEMTUZUMAB	ILARIS
	GEMZAR	APREMILAST
	GLEEVEC	OTEZLA
	GLIADEL	TOFACITINIB
	GOSERELIN	XELJANZ
	HERCEPTIN	BARICITINIB
	HEXALEN	OLUMIANT
	HYCAMTIN	UPADACITINIB
	HYDREA	RINVOQ
	HYDROXYUREA	FILGOTINIB
	INDIUM	JYSELECA
	YTTRIUM	
	IBRUTINIB	**III. Other Immunosuppressants**
	IMBRUVICA	
	IDAMYCIN	AFELIMOMAB
	IDARUBICIN	ALEFACEPT
	IFEX	AMEVIVE
	IFOSFAMIDE	AZASAN
	IMATINIB	AZATHIOPRINE
	INEBILIZUMAB	ANTITHYMOCYTE
	INLYTA	GLOBULIN
	INTERFERON	THYMOGLOBULIN
	INTRON	BASILIXIMAB
	IRESSA	BELIMUMAB
	IRINOTECAN	BENLYSTA
	IXABEPILONE	BELATACEPT
	IXAZOMIB	NULOJIX
	IXEMPRA	BRODALUMAB
	JAKAFI	SILIQ
	KESIMPTA	CELLCEPT
	KYPROLIS	CYCLOSPORINE
	LAPATINIB	DACLIZUMAB

© 2022 Akpandak I et al. *JAMA Network Open*.

Medication type	Medication name
	LEMTRADA<br>LENALIDOMIDE<br>LETROZOLE<br>LEUKERAN<br>LEUPROLIDE<br>LEUSTATIN<br>LEVAMISOLE<br>LEVULAN<br>LOMUSTINE<br>LONSURF<br>LUPRON<br>LYSODREN<br>MATULANE<br>MECHLORETHAMINE<br>MEGESTROL<br>MEKINIST<br>MELPHALAN<br>MERCAPTOPURINE<br>METHOTREXATE<br>METHOXSALEN<br>MITOMYCIN<br>MITOTANE<br>MITOXANTRONE<br>MUSTARGEN<br>MUTAMYCIN<br>MYLERAN<br>MYLOCEL<br>MYLOTARG<br>NAVELBINE<br>NELARABINE<br>NEOSAR<br>NEXAVAR<br>NILANDRON<br>NILOTINIB<br>NILUTAMIDE<br>NINLARO<br>NIPENT<br>NOLVADEX<br>NOVANTRONE<br>NPLATE<br>OFATUMUMAB<br>ONCASPAR<br>ONTAK<br>ONXOL<br>OXALIPLATIN<br>PACLITAXEL<br>PANITUMUMAB<br>PANRETIN<br>PARAPLATIN<br>PAZOPANIB<br>PEGASPARGASE<br>PEMETREXED<br>PENTOSTATIN<br>PHOTOFRIN<br>ZINBRYTA<br>DUPILUMAB<br>DUPLIXENT<br>ECULIZUMAB<br>SOLIRIS<br>EFALIZUMAB<br>RAPTIVA<br>EMAPALUMAB<br>GAMIFANT<br>GENGRAF<br>GOLIMUMAB<br>GUSELKUMAB<br>TREMFYA<br>SIMPONI<br>HYDROXYCHLOROQUINE<br>INTERFERON BETA<br>PEGINTERFERON BETA<br>REBIF<br>CINNOVEX<br>PLEGRIDY<br>BETAFERON<br>OCRELIZUMAB<br>OCREVUS<br>PLAQUENIL<br>IMURAN<br>MUROMONAB<br>MYCOPHENOLATE<br>MOFETIL<br>MYCOPHENOLIC ACID<br>MYFORTIC<br>NATALIZUMAB<br>TYSABRI<br>NEORAL<br>OMALIZUMAB<br>XOLAIR<br>ORTHOCLONE<br>PROGRAF<br>RAPAMUNE<br>RAPAMYCIN<br>RAVULIZUMAB<br>ULTOMIRIS<br>RILANOCEPT<br>ARCALYST<br>SATRALIZUMAB<br>ENSPRYNG<br>SANDIMMUNE<br>SIMULECT<br>SIROLIMUS<br>TACROLIMUS<br>TILDRAKIZUMAB<br>ILUMYA<br>VEDOLIZUMAB<br>ENTYVIO<br>ZENAPA

© 2022 Akpandak I et al. *JAMA Network Open*.

Medication type	Medication name	
	PLATINOL	IMMUNOGLOBULIN
	PLENAXIS	FINGOLIMOD
	PLICAMYCIN	GILENYA
	POMALIDOMIDE	TERIFLUNOMIDE
	PONATINIB	AUBAGIO
	PORFIMER	OZANIMOD
	PRALATREXATE	ZEPOSIA
	PROCARBAZINE	SIPONIMOD
	PROLEUKIN	MAYZENT
	PURINETHOL	DIMETHYL FUMARATE
	REGORAFENIB	GLATIRAMER ACETATE
	REVLIMID	COPAXONE
	RITUXAN	DIROXIMEL FUMARATE
	RITUXIMAB	MONOMETHYL
	ROFERON	FUMARATE
	ROMIDEPSIN	VOCLOSPORIN
	ROMIPLOSTIM	LUPKYNIS
	RUXOLITINIB	MEPOLIZUMAB
		NUCALA
		BENRALIZUMAB
		FASENRA
		RESLIZUMAB
		CINQAIR

© 2022 Akpandak I et al. *JAMA Network Open*.

eTable 8. *ICD-10* Codes and Descriptions Used to Identify COVID-19 Infection

COVID-19-related ICD-10 codes	Dates effective	Description
B97.29	Before 4/1/2020	Other coronavirus as the cause of diseases classified elsewhere
U07.1	4/1/2020	Coronavirus disease 2019, virus identified
J12.82	1/1/2021	Pneumonia due to coronavirus disease 2019

© 2022 Akpandak I et al. *JAMA Network Open*.

eTable 9. Negative Control Outcomes and Corresponding *ICD-10* Codes

**Condition**	**ICD-10 code(s)**
Kidney or ureteral stone	N20.x
Pyelonephritis	N10.x
Irritant contact dermatitis	L24.x

© 2022 Akpandak I et al. *JAMA Network Open*.

eTable 10. Characteristics of Cohort Study Population by Vaccine Exposure

**Characteristic**	**COVID-19 vaccinated**	**Influenza vaccinated pre-pandemic**	**Influenza vaccinated early pandemic**
**Total patients**	2 039 854 (100.0%)	5 058 394 (100.0%)	4 029 131 (100.0%)
**Age (years)**			
Mean (SD)	43.2 (16.3)	56.2 (20.6)	58.4 (20.0)
Median [Q1, Q3]	44.0 [31.0, 56.0]	61.0 [41.0, 72.0]	64.0 [45.0, 73.0]
**Gender**			
Female	1 031 149 (50.6%)	2 833 704 (56.0%)	2 273 801 (56.4%)
Male	1 008 543 (49.4%)	2 224 242 (44.0%)	1 755 063 (43.6%)
Unknown	162 (0.0%)	448 (0.0%)	267 (0.0%)
**Race/ethnicity**[a]			
Asian	139 826 (6.9%)	236 882 (4.7%)	193 362 (4.8%)
Black	141 582 (6.9%)	486 213 (9.6%)	364 603 (9.0%)
Hispanic	205 463 (10.1%)	455 555 (9.0%)	350 773 (8.7%)
White	1 344 318 (65.9%)	3 583 181 (70.8%)	2 844 543 (70.6%)
Unknown	208 665 (10.2%)	296 563 (5.9%)	275 850 (6.8%)
**Healthcare utilization**			
Ambulatory visit count			
Mean (SD)	8.35 (11.5)	13.8 (18.2)	12.3 (17.1)
Median [Q1, Q3]	5.00 [2.00, 10.0]	8.00 [4.00, 17.0]	7.00 [3.00, 15.0]
Inpatient visit ever	227 146 (11.1%)	1 013 821 (20.0%)	654 203 (16.2%)
**Medical history**			
Asthma	85 468 (4.2%)	336 105 (6.6%)	245 985 (6.1%)
Autoimmune disease	108 250 (5.3%)	466 190 (9.2%)	361 014 (9.0%)
Cancer	52 850 (2.6%)	346 308 (6.8%)	280 487 (7.0%)
Cardiovascular disease	65 735 (3.2%)	686 891 (13.6%)	544 163 (13.5%)
Chronic kidney disease	40 655 (2.0%)	496 602 (9.8%)	420 823 (10.4%)
Chronic lung disease	34 663 (1.7%)	485 942 (9.6%)	344 860 (8.6%)
Diabetes (any type)	154 694 (7.6%)	1 018 997 (20.1%)	808 358 (20.1%)
HIV/AIDS	5130 (0.3%)	19 405 (0.4%)	13 174 (0.3%)
Solid organ transplantation	3929 (0.2%)	22 214 (0.4%)	17 137 (0.4%)
Recent COVID-19 infection[b,c]	20 482 (1.0%)	N/A	58 472 (1.5%)
Received zoster vaccine	160 380 (7.9%)	604 390 (11.9%)	738 551 (18.3%)
**Medication use**[c]			
Systemic corticosteroids	10 788 (0.5%)	92 782 (1.8%)	63 158 (1.6%)
Other immunosuppressants	36 998 (1.8%)	154 243 (3.0%)	131 780 (3.3%)
Antivirals	14 300 (0.7%)	47 352 (0.9%)	36 439 (0.9%)

Abbreviations: HIV=human immunodeficiency virus; AIDS=acquired immunodeficiency syndrome.
[a] Due to rounding, the percentages for the influenza vaccinated early pandemic group sum to 99.9%.
[b] Recent COVID-19 infection: infection within 30 days prior to the start of the risk interval up to the event/censor date.
[c] For COVID-19 vaccinated patients, recent COVID-19 infection and medication use were measured at the dose-level; the status at the first recorded dose was used to report the frequency and percent in this table.

© 2022 Akpandak I et al. *JAMA Network Open*.

eTable 11. Risk of Herpes Zoster Infection Among COVID-19 Vaccinated vs Influenza Vaccinated, Prepandemic

Characteristic	HZ cases	Person-years	Unadjusted HR[a] (95% CI)	Adjusted HR[a] (95% CI)	p
**Vaccine type**					
Influenza 2018-2019	2686	415368.7	Reference[b]	Reference[b]	
1st dose COVID-19	459	139212.5	0.51 (0.46 - 0.56)	0.78 (0.70 - 0.86)	<0.001
2nd dose COVID-19	432	128888.5	0.52 (0.47 - 0.57)	0.79 (0.71 - 0.88)	<0.001
**Age (years)**	--	--	1.03 (1.03 - 1.03)	1.026 (1.024 - 1.027)	<0.001
**Gender**					
Female	2208	368651.9	Reference[b]	Reference[b]	
Male	1369	314760.5	0.73 (0.68 - 0.78)	0.77 (0.72 - 0.82)	<0.001
Unknown	0	57.3	Not estimable	Not estimable	
**Race/ethnicity**					
Asian	168	37944.9	0.80 (0.69 - 0.94)	1.05 (0.90 - 1.23)	0.52
Black	289	58322.6	0.90 (0.80 - 1.01)	0.76 (0.67 - 0.86)	<0.001
Hispanic	327	64213.9	0.92 (0.82 - 1.04)	1.00 (0.89 - 1.12)	0.99
White	2599	471487.1	Reference[b]	Reference[b]	
Unknown	194	51501.2	0.68 (0.59 - 0.79)	0.91 (0.79 - 1.06)	0.23
**Healthcare utilization**					
Ambulatory visit count	--	--	1.01 (1.01 - 1.01)	1.004 (1.002 - 1.005)	<0.001
Inpatient visit ever	840	112870.6	1.55 (1.44 - 1.68)	1.09 (1.00 - 1.19)	0.06
**Medical history**					
Asthma	269	38862.2	1.35 (1.19 - 1.53)	1.12 (0.99 - 1.27)	0.08
Autoimmune disease	464	52597.8	1.79 (1.62 - 1.97)	1.25 (1.12 - 1.39)	<0.001
Cancer	327	35418.5	1.84 (1.64 - 2.06)	1.12 (0.99 - 1.26)	0.07
Cardiovascular disease	602	65019.7	1.92 (1.76 - 2.1)	1.05 (0.95 - 1.17)	0.30
Chronic kidney disease	366	46049.3	1.58 (1.42 - 1.76)	0.77 (0.68 - 0.87)	<0.001
Chronic lung disease	448	44403.9	2.06 (1.87 - 2.27)	1.14 (1.03 - 1.27)	0.02
Diabetes (any type)	779	104087.7	1.55 (1.43 - 1.68)	1.02 (0.93 - 1.11)	0.68
HIV/AIDS	12	2266.9	1.01 (0.57 - 1.78)	1.27 (0.72 - 2.24)	0.41
Solid organ transplantation	33	2339.4	2.71 (1.92 - 3.82)	1.80 (1.26 - 2.57)	0.001
Received zoster vaccine	255	71115.3	0.66 (0.58 - 0.75)	0.44 (0.38 - 0.50)	<0.001
**Medication use**					
Systemic corticosteroids	120	9025.7	2.59 (2.16 - 3.11)	1.51 (1.24 - 1.83)	<0.001
Other immunosuppressants	190	17581.9	2.12 (1.84 - 2.46)	1.29 (1.10 - 1.51)	0.002
Antivirals	15	5781.5	0.49 (0.30 - 0.82)	0.41 (0.25 - 0.69)	0.001

Abbreviations: HZ=herpes zoster; HR=hazard ratio, CI=confidence interval; HIV=human immunodeficiency virus; AIDS=acquired immunodeficiency syndrome.

[a] HRs, p-values, and 95% CIs calculated using Cox proportional hazards regression with robust standard errors clustered on individuals. P-values displayed correspond to adjusted HRs. The adjusted models include all variables listed in the table.

[b] Influenza-vaccinated individuals vaccinated pre-pandemic served as the reference group to calculate the hazard ratios of HZ in the COVID-19 vaccinated group. Female individuals and White individuals served as the reference groups for gender and race/ethnicity, respectively. For comorbidities, inpatient visits, and medications, individuals without a history of the condition, visit, or use of the medication served as the reference group for calculating the hazard ratios for those exposures.

© 2022 Akpandak I et al. *JAMA Network Open*.

eTable 12. Risk of Herpes Zoster Infection Among COVID-19 Vaccinated vs Influenza Vaccinated, Early Pandemic

Characteristic	HZ cases	Person-years	Unadjusted HR[a] (95% CI)	Adjusted HR[a] (95% CI)	p
**Vaccine type**					
Influenza 2020	1899	330860.1	Reference[b]	Reference[b]	
1st dose COVID-19	459	139212.5	0.57 (0.52 - 0.64)	0.89 (0.80 - 1.00)	0.05
2nd dose COVID-19	432	128888.5	0.58 (0.53 - 0.65)	0.91 (0.81 - 1.02)	0.09
**Age (years)**	--	--	1.027 (1.025 - 1.029)	1.027 (1.024 - 1.029)	<0.001
**Gender**					
Female	>1696	322683.7	Reference[b]	Reference[b]	
Male	1083	276234.9	0.74 (0.69 - 0.80)	0.78 (0.72 - 0.84)	<0.001
Unknown	<11	42.4	4.46 (0.63 - 31.69)	5.09 (0.72 - 36.12)	0.10
**Race/ethnicity**					
Asian	152	34371.2	0.90 (0.77 - 1.06)	1.17 (0.99 - 1.39)	0.06
Black	213	48337.7	0.90 (0.78 - 1.04)	0.76 (0.66 - 0.88)	<0.001
Hispanic	263	55609.2	0.97 (0.85 - 1.10)	1.02 (0.90 - 1.17)	0.72
White	2013	410841.5	Reference[b]	Reference[b]	
Unknown	149	49801.4	0.61 (0.52 - 0.72)	0.82 (0.69 - 0.97)	0.02
**Healthcare utilization**					
Ambulatory visit count	--	--	1.011 (1.010 - 1.012)	1.005 (1.004 - 1.007)	<0.001
Inpatient visit ever	562	83343.6	1.56 (1.42 - 1.71)	1.12 (1.01 - 1.24)	0.03
**Medical history**					
Asthma	198	31462.9	1.38 (1.19 - 1.59)	1.17 (1.01 - 1.35)	0.04
Autoimmune disease	326	43964.4	1.67 (1.49 - 1.87)	1.15 (1.01 - 1.31)	0.03
Cancer	257	30014.9	1.92 (1.69 - 2.19)	1.16 (1.02 - 1.33)	0.03
Cardiovascular disease	453	53302	1.98 (1.79 - 2.19)	1.09 (0.97 - 1.23)	0.13
Chronic kidney disease	322	39826	1.83 (1.63 - 2.06)	0.91 (0.80 - 1.04)	0.17
Chronic lung disease	275	32822.4	1.88 (1.66 - 2.13)	1.01 (0.88 - 1.16)	0.86
Diabetes (any type)	588	86793.6	1.57 (1.44 - 1.73)	1.02 (0.92 - 1.13)	0.71
HIV/AIDS	<11	1755.3	0.73 (0.33 - 1.63)	0.87 (0.39 - 1.95)	0.74
Solid organ transplantation	23	1922.8	2.58 (1.71 - 3.89)	1.45 (0.94 - 2.24)	0.09
Received zoster vaccine	249	82134.8	0.62 (0.54 - 0.70)	0.39 (0.34 - 0.45)	<0.001
Recent COVID-19 infection[c]	30	6996	0.92 (0.64 - 1.32)	0.75 (0.52 - 1.08)	0.12
**Medication use**					
Systemic corticosteroids	77	6594	2.55 (2.03 - 3.20)	1.45 (1.14 - 1.85)	0.003
Other immunosuppressants	153	15738.6	2.15 (1.83 - 2.53)	1.36 (1.13 - 1.64)	0.001
Antivirals	26	4884.6	1.14 (0.78 - 1.68)	0.97 (0.65 - 1.44)	0.88

Abbreviations: HZ=herpes zoster; HR=hazard ratio, CI=confidence interval; HIV=human immunodeficiency virus; AIDS=acquired immunodeficiency syndrome.

[a] HRs, p-values, and 95% CIs calculated using Cox proportional hazards regression with robust standard errors clustered on individuals. P-values displayed correspond to adjusted HRs. The adjusted models include all variables listed in the table.

[b] Influenza-vaccinated individuals vaccinated early in the pandemic served as the reference group to calculate the hazard ratios of HZ in the COVID-19 vaccinated group. Female individuals and White individuals served as the reference groups for gender and race/ethnicity, respectively. For comorbidities, inpatient visits, and medications, individuals without a history of the condition, visit, or use of the medication served as the reference group for calculating the hazard ratios for those exposures.

[c] Recent COVID-19 infection: infection within 30 days prior to the start of the risk interval up to the event/censor date.

© 2022 Akpandak I et al. *JAMA Network Open*.

eTable 13. Comparison of Adjusted Hazard Ratios of Vaccination Type (COVID-19 vs Prepandemic Influenza and COVID-19 vs Early Pandemic Influenza) for Herpes Zoster and 3 Negative Control Outcomes

**Condition**	**Adjusted HR[a] (95% CI)** COVID-19 vs. pre-pandemic influenza		**Adjusted HR[a] (95% CI)** COVID-19 vs. early pandemic influenza	
	1st dose[b]	2nd dose[c]	1st dose[d]	2nd dose[e]
Herpes zoster	0.78 (0.70 - 0.86)	0.79 (0.71 - 0.88)	0.89 (0.80 - 1.00)	0.91 (0.81 - 1.02)
Kidney or ureteral stone	0.85 (0.81 - 0.88)	0.91 (0.87 - 0.95)	0.87 (0.83 - 0.91)	0.94 (0.90 - 0.98)
Pyelonephritis	0.72 (0.60 - 0.85)	0.68 (0.57 - 0.83)	0.87 (0.73 - 1.05)	0.84 (0.69 - 1.02)
Irritant contact dermatitis	0.89 (0.80 - 0.99)	1.01 (0.91 - 1.12)	0.89 (0.79 - 0.99)	1.01 (0.91 - 1.13)

Abbreviations: HR=hazard ratio, CI=confidence interval.
[a] HRs and 95% CIs calculated using Cox proportional hazards regression. Models were adjusted for the same variables adjusted for in the main cohort analyses.
[b] Hazard ratios in this column compare hazards of the given condition in individuals who received 1st dose of COVID-19 vaccine to individuals who received an influenza vaccine in the pre-pandemic period.
[c] Hazard ratios in this column compare hazards of the given condition in individuals who received 2nd dose of COVID-19 vaccine to individuals who received an influenza vaccine in the pre-pandemic period.
[d] Hazard ratios in this column compare hazards of the given condition in individuals who received 1st dose of COVID-19 vaccine to individuals who received an influenza vaccine in the early pandemic period.
[e] Hazard ratios in this column compare hazards of the given condition in individuals who received 2nd dose of COVID-19 vaccine to individuals who received an influenza vaccine in the early pandemic period.

© 2022 Akpandak I et al. *JAMA Network Open*.

eReferences

1. Centers for Medicare & Medicaid Services (CMS). *Assessing the Completeness of Medicare Claims Data for Measuring COVID-19 Vaccine Administration*. Centers for Medicare & Medicaid Services (CMS) Accessed March 22, 2022. https://www.cms.gov/files/document/assessing-completeness-medicare-claims-data-measuring-covid-19-vaccine-administration.pdf

2. Lam NN, Fleet JL, McArthur E, Blake PG, Garg AX. Higher dose versus lower dose of antiviral therapy in the treatment of herpes zoster infection in the elderly: a matched retrospective population-based cohort study. *BMC Pharmacol Toxicol*. 2014;15:48. doi:10.1186/2050-6511-15-48

© 2022 Akpandak I et al. *JAMA Network Open*.
